# The Positive Relationships of Playfulness With Indicators of Health, Activity, and Physical Fitness

**DOI:** 10.3389/fpsyg.2018.01440

**Published:** 2018-08-14

**Authors:** René T. Proyer, Fabian Gander, Emma J. Bertenshaw, Kay Brauer

**Affiliations:** ^1^Personality and Assessment, Department of Psychology, Martin-Luther University of Halle-Wittenberg, Halle, Germany; ^2^Personality and Assessment, Department of Psychology, University of Zürich, Zurich, Switzerland; ^3^Unilever R&D, Colworth Science Park, Bedford, United Kingdom

**Keywords:** adult playfulness, playfulness, health, activity, physical fitness, health behavior, OLIW, peer-ratings

## Abstract

Adult playfulness is a personality trait that enables people to frame or reframe everyday situations in such a way that they experience them as entertaining, intellectually stimulating, or personally interesting. Earlier research supports the notion that playfulness is associated with the pursuit of an active way of life. While playful children are typically described as being active, only limited knowledge exists on whether playfulness in adults is also associated with physical activity. Additionally, existing literature has not considered different facets of playfulness, but only global playfulness. Therefore, we employed a multifaceted model that allows distinguishing among *Other-directed, Lighthearted, Intellectual*, and *Whimsical* playfulness. For narrowing this gap in the literature, we conducted two studies addressing the associations of playfulness with health, activity, and fitness. The main aim of Study 1 was a comparison of self-ratings (*N* = 529) and ratings from knowledgeable others (*N* = 141). We tested the association of self- and peer-reported playfulness with self- and peer-reported physical activity, fitness, and health behaviors. There was a good convergence of playfulness among self- and peer-ratings (between *r* = 0.46 and 0.55, all *p* < 0.001). Data show that both self- and peer-ratings are differentially associated with physical activity, fitness, and health behaviors. For example, self-rated playfulness shared 3% of the variance with self-rated physical fitness and 14% with the pursuit of an active way of life. Study 2 provides data on the association between self-rated playfulness and objective measures of physical fitness (i.e., hand and forearm strength, lower body muscular strength and endurance, cardio-respiratory fitness, back and leg flexibility, and hand and finger dexterity) using a sample of *N* = 67 adults. Self-rated playfulness was associated with lower baseline and activity (climbing stairs) heart rate and faster recovery heart rate (correlation coefficients were between −0.19 and −0.24 for global playfulness). Overall, Study 2 supported the findings of Study 1 by showing positive associations of playfulness with objective indicators of physical fitness (primarily cardio-respiratory fitness). The findings represent a starting point for future studies on the relationships between playfulness, and health, activity, and physical fitness.

## Introduction

There is an agreement in the literature that play behaviors serve important functions in numerous developmental processes in infancy and childhood (see e.g., Bruner et al., 1976; Burghardt, 2005). For example, for animals it has been argued that play at a young age may help, for example, in muscle development and facilitate physical balance (e.g., Fagen, 1981). Playfulness as a *personality trait* in adults has hitherto been widely neglected in research and practice across disciplines. In particular, its correlates with variables of physical functioning in adult life are widely unknown. Nevertheless, a growing number of studies exist that support the notion that playfulness (the personality trait associated with play as the actual behavior) may serve an important role in several life domains of adults too. Amongst others, it has been shown that playfulness relates to positive outcome variables such as coping (e.g., Staempfli, 2007; Magnuson and Barnett, 2013), work performance and innovative behavior at work (Glynn and Webster, 1992; Yu et al., 2007), creativity and intrinsic motivation (Amabile et al., 1994; Proyer, 2012b), virtuousness (Proyer and Ruch, 2011), sexual selection (Chick et al., 2012; Proyer and Wagner, 2015), academic success (Proyer, 2011), low expressions in the Impostor phenomenon (Brauer and Proyer, 2017), or subjective well-being (Proyer, 2013, 2014a,b; Proyer et al., 2018a). The present study aims at extending these findings to health, activity, and physical fitness.

While there is no agreement in the literature about a definition of playfulness in adults as a personality trait, the recent years have seen an increase in the study of the variable. It has been argued that research has partially suffered from the usage of conceptualizations and assessment instruments that have failed to clearly differentiate between the core of playfulness and its consequences (e.g., when using statements such as “I laugh a lot” for the study of individual differences in *playfulness*; Proyer, 2012a; Proyer and Jehle, 2013), a lack of distinctiveness from related traits (e.g., humor, creativity, or curiosity; e.g., Proyer, 2018), and unwanted overlap with basic personality traits (mostly extraversion and emotional stability; Proyer and Jehle, 2013). Based on a mixed-methodology (e.g., psycho-linguistic, psychometric, and qualitative approaches; for an overview see Proyer, 2017) a new definition that aims at focusing on the core characteristics of playfulness has been proposed; namely,

“Playfulness is an individual differences variable that allows people to frame or reframe everyday situations in a way such that they experience them as entertaining, and/or intellectually stimulating, and/or personally interesting. Those on the high end of this dimension seek and establish situations in which they can interact playfully with others (e.g., playful teasing, shared play activities) and they are capable of using their playfulness even under difficult situations to resolve tension (e.g., in social interactions, or in work type settings). Playfulness is also associated with a preference for complexity rather than simplicity and a preference for—and liking of—unusual activities, objects and topics, or individuals” (Proyer, 2017 p. 114).

Hence, it is argued that playfulness in adults also contributes to other life domains (e.g., intellectual achievements, or personal involvement)—rather than entertainment alone, which has been highlighted as its main function in earlier definitions (e.g., Murray, 1938; Glynn and Webster, 1992; Barnett, 2007). A playful attitude can also help to gain a new perspective on serious topics and assist in coping with adverse circumstances (cf. the notion of serious-cheerfulness as one pursuit of the *homo ludens* in Rahner, 1948/2008; see also Proyer and Rodden, 2013; Proyer, 2014a). On a more descriptive level, playful people are typically seen as funny, humorous, spontaneous, unpredictable, active, energetic, adventurous, convivial, and cheerful and tend to display playful behavior by telling jokes, playing pranks, and horsing around (Barnett, 2007, 2011). In line with these descriptions, playfulness at a younger age has also been linked to greater physical activity or physical spontaneity (e.g., coordinated movements; Lieberman, 1977; see also Barnett, 1991; Singer et al., 1980).

Proyer (2017) proposes a new structural model of adult playfulness that differentiates among four facets; namely, (a) *O*ther-directed, (b) *L*ighthearted, (c) *I*ntellectual, and (d) *W*himsical playfulness (OLIW-model; see Table 1 for a description). Additionally, previous research has shown that playfulness could also be conceptualized and measured on a global level (playfulness in general), in terms of an easy onset and high intensity of playful experiences along with the frequent display of playful activities (Proyer, 2012a). We will use both a global assessment of playfulness and a measure for the four facets for a more fine-grained differentiation of the variable.

**Table 1:** Description of the different playfulness facets.

**Trait**	**Description**
Global Playfulness	Global Playfulness is an individual differences variable that allows people to frame or reframe everyday situations in a way such that they experience them as entertaining, and/or intellectually stimulating, and/or personally interesting.
Other-directed	The facet of other-directed playfulness is characterized by the use of playful behaviors in social situations. High scorers use playfulness to ease tense situations, and cheer other people up, they enjoy horsing around with friends and engage, generally, in a playful interaction style with other people
Lighthearted	The facet of lighthearted playfulness is characterized by a spontaneous, carefree view of life. High scorers do not think much about possible consequences of their behavior but prefer and enjoy improvising in comparison with elaborate preparation.
Intellectual-Creative	The facet of intellectual playfulness is characterized by the enjoyment of playing with ideas. High scorers like to puzzle over problems and to come up with new, creative solutions for problems.
Whimsical	The facet of whimsical playfulness is characterized by a preference for breaking ranks. High scorers are amused by oddities and have a preference for extraordinary things and people. Others often regard them as extravagant.

A particularly understudied topic in the research on adult playfulness is the study of physical activity and fitness. It has been suggested that play in children might serve as a practice for future skills (although this idea has also been disputed; see Chick, 2001; Burghardt, 2005). A first hint on a positive association between playfulness and physical health, greater activity, and fitness comes from Lieberman's (1977) early work on playfulness in children. For adults, she argues that “[…] through its component parts of sense of humor, manifest joy, and spontaneity, it has major implications for childrearing practices, educational planning, career choices, and leisure pursuits” (Lieberman, 1977 p. xi) and later she notes “[…] playfulness as a quality of play would developmentally transform itself into a personality trait of the player in adolescence and adulthood“ (Lieberman, 1977, p. 23).

### Associations between playfulness, health, activity, and fitness

There are few published studies exploring the relationship between playfulness and indicators of health, activity, and fitness. Therefore, the present studies aim to extend and build upon limited knowledge of the relationships. Existing literature and theoretical reasoning allow us to derive an initial framework that supports the notion of an association. There are several ways to think about the potential link between adult playfulness and markers of physical functioning: Firstly, the association may be developed *indirectly via positive affect*: Playful personalities give rise to feeling playful more frequently which is associated itself with experiencing a pleasant, positive feeling. Positive affect is associated with successful health outcomes and behaviors in some studies, although the evidence is mixed in general (e.g., Lyubomirsky et al., 2005; Cameron et al., 2017). Accordingly, Fredrickson (1998, 2001) argues that positive emotions may contribute to physical resources such as coordination, strength, or cardiovascular health by strengthening personal resources (e.g., via joint social activities that elicit positive emotions and that may consequently also result in greater levels of activities)^1^. The experienced positive affect may also buffer against (emotional) distress that may be a hindering factor for engaging in physical activity or limiting a persons' desire to actively engage with their environment. Secondly, a positive association between playfulness and physical functioning can also manifest as *action orientated tendencies* toward curiosity, exploration, or physical activities; playful people may be interested in trying and doing a greater range of activities. This notion receives support from studies showing that those high in playfulness also seem to have an active interest in the pursuit of leisure time activities (e.g., Mannell, 1984), experience low boredom in their leisure time (e.g., Barnett, 2011), have good skills to cope with adversities and possess a mastery orientation (e.g., Staempfli, 2007; Magnuson and Barnett, 2013; Proyer, 2014a) and have an interest in the pursuit of enjoyable activities (e.g., doing fun things with others or communing with nature; Proyer, 2013).

While a full analysis of motivational factors associated with playfulness is missing, there are data linking greater expressions of playfulness with intrinsic motivation (Amabile et al., 1994; Proyer, 2012b). Given that many models which describe the structure of adult playfulness, contain other-directed facets (e.g., Lieberman, 1977; Barnett, 2007; Proyer, 2017) one might assume that affiliation may also be related to playfulness. This may help playful adults not only to engage in team sports, but also in other joint activities with others (e.g., outdoor activities) and, in general, be associated with greater engagement with one's environment. Playfulness may facilitate achievement in active sports as it has been linked with intrinsic motivation and motivation toward achievement (see e.g., Proyer, 2011, 2012b), innovation and creativity (e.g., Yu et al., 2007; Bateson and Martin, 2013; for an overview see Proyer et al., 2018b), and competitiveness (see Csikszentmihalyi, 1975) to name but a few.

Thirdly, those high in playfulness may be in general more interested in *promoting their own health*. For example, Proyer (2013) found positive associations between playfulness and health-behaviors such as pursuing a more active way of life. Such activities may then contribute positively to an individual's health. However, playfulness also showed some negative relationships with specific health behaviors, such as security orientation (e.g., wearing a safety belt in the car or avoiding violence), substance use (e.g., drinking coffee or alcohol), and hygiene (e.g., regularly brushing teeth, or using floss). This may be associated with a certain lighthearted attitude (Proyer, 2017) in dealing with the daily life and speaks for the need to differentiate between facets of playfulness and different indicators of physical functioning. Fourthly, playfulness may either directly or indirectly have a sustained effect on the development of health via skills needed for physical functioning, based on mechanisms not identified above. It is possible that genetic predispositions interact with trait playfulness to increase physical activity. Another example could be the choice of vocational activity that may be influenced by the fit between a person's trait playfulness and his/her preferred activities. Proyer (2013) showed that global playfulness goes along with better (physical) coordination skills (Proyer, 2013; *N* = 255, self-reported data in a correlational study). In this sense, playfulness may facilitate greater physical activity as those higher in playfulness have greater skills in certain areas (e.g., coordination) and use them accordingly (e.g., in sports, or other activities that require greater skill-levels), or in the sense that playfulness supports the development and acquisition of certain health-related skills.

Taken together these findings speak for greater levels of activity among highly playful people. Thus, playfulness might affect health and physical skills through increased activity. On the other hand, highly playful people may be more likely to be physically active because they aim for greater physical and or mental health. The relation between playfulness, health, activity, and fitness is likely to be complex, so in these studies we focus on testing particular associations between personality and physical activity. Since playfulness is assumed to be a multi-dimensional construct, the playful facets [e.g., playfulness in its *Other-directed* (e.g., team sport, group activities) or *Lighthearted* (e.g., being open for new activities) facet] may be more strongly correlated with different indicators of health, activity, and fitness than a single global score that averages playfulness across different domains. Also, the findings so far relied on self-reports for both playfulness and indicators of health, activity, and fitness and it is unclear to what extent these results are due to a shared method influence. The problem in this case is that “[…] one has no way of distinguishing trait variance from unwanted method variance” (Campbell and Fiske, 1959, p. 102). One possible solution is the consideration of additional methods and testing whether the findings are comparable.

We aim to expand the validity of the findings by additionally collecting peer-ratings of a well-acquainted informant for each participant, on both the playfulness and indicators of health variables. This approach has two main merits: Firstly, there is a broad range of research that has shown that although self-ratings provide a valid source of information, self-perceptions are prone to biases (cf. Connolly and Ones, 2010). For example, Vazire and Mehl (2008) have shown that peer-ratings are not only as accurate as self-ratings, but that they *independently* predict behavioral outcomes and provide a unique insight. Hence, we aim to implement to collect peer-ratings for each tested participant. Moreover, we follow Hofstee's (1994) approach to aggregate the self- and peer-rating to provide an approximation of the participants “true” personality. We will use this for studying associations of playfulness with health, activity, and fitness. Secondly, this approach allows reducing common method variance (Campbell and Fiske, 1959) based on the same assessment techniques (i.e., questionnaires).

The present set of studies aimed at replicating and extending previous findings (e.g., Proyer, 2013) in several ways. Study 1 uses an online survey methodology and extends previous findings by considering a broad array of indicators of health, activity, fitness; studying different facets of playfulness, in addition to global playfulness. Further, we collected informant ratings of well-acquainted peers for playfulness and health indicators. This approach allows us to test whether the correlational patterns are comparable for the same variable in self- and peer-ratings with other variables. Study 2 uses objective indicators of health, activity, and fitness, as well as playfulness (as measured in a laboratory setting) for examining their associations in a methodologically more rigorous approach, in order to overcome the shortcomings of having to rely on self-ratings only.

## Study 1

Study 1 examines the relationships of self-and peer-rated global playfulness and its facets with mental and physical health (self-ratings) as well as physical activity, physical fitness, and health-behaviors (self- and peer-ratings). Based on earlier findings (Proyer, 2013), we expected positive relationships of playfulness with self-rated mental health and selected health behavior such as leading an active way of life, but also substance use. In line with Proyer (2013), we expected negative relationships with compliance (e.g., taking care and complying to regulations in road traffic), but positive associations with levels of activity and physical health. On the level of specific facets, we expected the largest relationships for *Other-directed* playfulness, since this aspect has the strongest conceptual overlap with being active and engaging in (play) activities with others in comparison to other, more cognitive aspects of playfulness. We assume that there are indirect effects of having more contact with others and either inviting them to join an own activity or participating in theirs. This type of playful exchange with others should be particularly helpful in engaging in activities. Of course, not all of these will be positively associated with health and physical fitness but leading a more active way of life should also include physical activities. The other facets are expected to show comparatively smaller or no relationships with health, fitness, and well-being.

Finally, we expected these relationships to be present in all data sources (i.e., when looking at relationships between self-rated playfulness and self-rated health/activity/fitness, but also self- and peer-ratings, peer- and self-ratings, and peer- and peer-ratings). Previous studies found that self- and peer-rated playfulness converges in a range of 0.44–0.57 across the four OLIW-facets in a mixed sample of highly acquainted people (good friends, romantic partners, siblings, etc., Proyer, 2017), between 0.33 and 0.58 in heterosexual couples in a romantic relationship (Proyer et al., 2018a), and between 0.21 and 0.37 in a zero-acquaintance setting (ratings of short self-descriptions of up to five sentences; Proyer and Brauer, 2018). Thus, the convergence was within the range which has been reported for other personality traits. For example, Funder et al. (1995) reported for facets of the big five an average correlation of *r* = 0.37 between self- and parents' ratings, and relationships of *r* = 0.36 and *r* = 0.30 when testing college and hometown acquaintances, respectively. We expect overlap in the range of these coefficients in the present study. We also expect robust convergence among self-and peer-ratings for our measures of activity, fitness, and health behaviors. However, it must be noted that some of these may be more difficult to observe than others. It has been argued that personality traits differ in their observability/evaluativeness (see Vazire, 2010) and similarly, depending on the level of acquaintance, some health behaviors or pursued activities may be low in observability. Moreover, we will test the overlap between the observed relationships of self- and peer-rated playfulness with activity, fitness, and health behaviors by computing the vector correlations (see Borkenau and Liebler, 1992) for each playfulness facet. This approach allows to estimate the overall overlap of the observed correlations when comparing the self- vs. peer ratings.

### Methods

#### Sample

A total of *N* = 529 participants (81.1% women) aged 18–78 years (*M* = 37.41, *SD* = 15.90) took part in an online survey. A breakdown into age-categories shows that a large portion of participants were between 18 and 29 years old (43.5%), 10.8% were between 30 and 39, 20.6% were between 40 and 49, 15.3% were between 50 and 59, and 9.8% were 60 years or older. A large part of the sample (41.6%) had a degree from a university or a university of applied sciences (BSc or higher), 36.1% held a degree allowing them to attend a university or a university of applied sciences, 16.3% completed vocational training, 5.8% have completed mandatory school, and only one participant (0.2%) had not completed mandatory school. Most participants (50.3%) were Swiss, or German (43.2%). More than half of the participants were currently in a relationship (60.3%), 33.3% were single, 5.1% were divorced/separated, and 1.3% were widowed. Most participants were currently employed (67.5%). Overall, the sample is diverse regarding age and other demographic characteristics.

About a fourth of the sample (141 participants; 26.7%) also provided peer-reports of the playfulness measures. The peer-raters were mostly female (53.9%) and aged 17 to 83 (*M* = 39.78, *SD* = 14.94). The largest portion of peer-raters was the romantic partner of the person who provided the self-report (48.9%), a family member (27.7%), or a (close) friend (23.4%).

#### Instruments

##### Playfulness measures

The *Short Measure of Adult Playfulness* (SMAP; Proyer, 2012a) assesses global playfulness with five items on a 7-point scale (1 = “does not apply at all” to 7 = “applies completely”). A sample item is “I am a playful person.” Internal consistency was high (α = 0.90).

The *OLIW-playfulness questionnaire* (OLIW; Proyer, 2017) assesses the four facets of playfulness (*Other-directed, Lighthearted, Intellectual*, and *Whimsical* playfulness) with seven items each on a 7-point scale (1 = “does not apply at all” to 7 = “applies completely”). A sample item is “I can use my playfulness to bring joy to other people or cheer them up” (*Other-directed* playfulness). Internal consistencies ranged from α = 0.66 (*Intellectual*) to α = 0.77 (*Whimsical*).

##### Health, activity, and fitness measures

Single items for the assessment of *Subjective Physical and Mental Health*; assessed with one item each, ranging from 0 (= “very bad”) to 10 (= “very good”). Single-item ratings of the subjective health status have been reported to be stable (Miilunpalo et al., 1997) and substantially related to external criteria such as number of physician visits (Miilunpalo et al., 1997), or mortality (for an overview see Idler and Benyamini, 1997).

The *General Level of Activity* (GLA; Proyer et al., 2014) was assessed with four single items for the comparison of (a) the own activity level in general (“I would consider myself a not very active person” vs. “a very active person”); (b) the own activity level in comparison with the person's peers (“Compared to other persons of my age and gender I would consider myself a not very active person” vs. “a very active person”); (c) comparison with people that are generally very active (“Some people are very active. They try to be active whenever possible and are looking for ways to complete tasks in a way that involves movement and physical activity. To what extent does this describe you?”); and (d) comparison with people that are generally not very active (“Some people are not very active. Although they are not lazy, they are never as active as they could be. To what extent does this describe you?” [recoded item]). All items are answered on a 7-point scale (1 = “not at all” to 7 = “to a very large extent”). Internal consistency was high (α = 0.89).

The *International Physical Activity Questionnaire* long-form (IPAQ; Craig et al., 2003) asks about the time spent (i.e., minutes per week) with physical activity in the past 7 days in four domains (work, active transportation, domestic and garden, and leisure-time). The questionnaire distinguishes among activities of low, moderate, and vigorous intensity. The IPAQ allows calculating specific scores for the four domains, and a total score. Additionally, scores for the time spent sitting, and the time spent in transportation (“passive transportation”) can be computed. Craig et al. (2003) report acceptable measurement properties, while moderate validity of the German long form in comparison with objective data was reported (Wanner et al., 2016).

The *Physical Fitness Questionnaire* (FFB-MOT; Bös et al., 2002) asks for the ease of performing twelve physical exercises. The FFB-MOT assesses general physical fitness in four basic motor abilities and a total score (“global fitness”): Cardiorespiratory fitness (e.g., “running one kilometer without a break”), strength (e.g., “carrying a heavy basket [8kg] over several floors”), flexibility (e.g., “tying shoelaces while standing upright”), and coordination (e.g., “doing a somersault”). Items are rated on a five-point scale (1 = “I cannot perform this exercise” to 5 = “I can perform this exercise without any problems”). Internal consistency was α = 0.82 for the total score and ranged from α = 0.66 (coordination) to α = 0.85 (cardiorespiratory fitness) for the basic abilities.

The Multiple Health Behavior Questionnaire (MHB-39; Wiesmann et al., 2003) assesses the frequency of performing 39 health-related behaviors on a five-point scale (1 = “*never*” to 5 = “*always*”). The internal consistency of the total scale was high (α = 0.83). Additionally, Wiesmann et al. (2003) and Proyer et al. (2013) extracted six orthogonal factors in a principal component analysis. These factors were labeled: Active way of life (e.g., being frequently physically active), compliance (e.g., visiting a physician when becoming aware of physical symptoms), substance use (e.g., drinking coffee or alcohol), security orientation (e.g., wearing a seatbelt in the car), diet (e.g., eating sweet dishes), and hygiene (e.g., using dental floss). For comparison purposes across studies and self- and peer-ratings, we used the regression weights from Proyer et al. (2013) to replicate their factor solution in this study.

#### Procedure

Participants were recruited through online-advertisements (forums, mailing lists), and by contacting participants from earlier (unrelated) studies. All participants gave informed consent and completed all instruments online. After the end of the survey, participants were asked to forward the link of the online study to a person who knows them well (no further restrictions/inclusion criteria were implemented). These people were asked to complete peer-ratings of the SMAP, the OLIW, and the GLA, the FFB-MOT, and the MHB-39.

Participants were not financially compensated but received an automated feedback on their individual scores upon completion of the study and had the opportunity to enter a prize draft for one of 10 online shopping vouchers worth 25 Swiss Francs. Peer-raters did not receive any incentive for participation.

### Results

In a first step, we examined the convergence of self- with peer-rated measures (see Supplementary Table A). Overall, the self-peer convergence was high for playfulness and its facets (all *r*s >0.46), while all other relationships outside the main axis were smaller in size (all *r*s ≤ 0.34). We also examined the agreement among self- and peer-ratings for activity, fitness, and health behaviors. Again, the relationships were high and ranged between *r* = 0.47 and *r* = 0.74 (mean = 0.68), while the correlations between the same constructs (i.e., the correlations in the main axis) were numerically higher than those between different constructs (see Supplementary Table B for all coefficients). Thus, self- and peer-ratings converged very well and were in the expected range for all measures.

Further correlational analyses (not reported in full detail) revealed small relationships with gender (men showed higher scores in global [*r* = −0.10] and *Lighthearted* playfulness [*r* = −0.11]) and small to medium-sized relationships with age (global [*r* = −0.10] and *Other-directed* playfulness [*r* = −0.28] showed negative relationships with age, while *Lighthearted* [*r* = 0.16], *Intellectual* [*r* = 0.20], and *Whimsical* [*r* = 0.14] playfulness were positively related to age). Therefore, we controlled for the influence of gender and age in all main analyses.

In a next step, we examined the relationships of playfulness with health, activity, and fitness. First, we present the associations of self- and peer-rated playfulness with *self-rated* indicators of health, activity, and fitness (Table 2), and afterwards the associations of self- and peer-rated playfulness with *peer-rated* indicators (Table 3), and finally the relationships between averaged self- and peer-ratings of playfulness and the indicators of health, activity, and fitness (Supplementary Table C).

**Table 2:** Relationships of self- and peer-rated playfulness with different self-rated indicators of health, activity, and fitness, controlled for gender and age.

**Self-ratings**	**Self**						**Peer**					
	**SMAP**	**OTD**	**LTH**	**INT**	**WHI**	***R*^2^ (OLIW)**	**SMAP**	**OTD**	**LTH**	**INT**	**WHI**	***R*^2^ (OLIW)**
Mental health	0.04	0.14^**^	0.26^***^	0.14^**^	−0.02	0.09^***^	0.10	0.14	0.13	0.13	0.01	0.03
Physical health	0.02	0.04	0.17^***^	0.06	0.01	0.03^**^	0.07	0.08	0.06	0.10	−0.04	0.02
Global activity level (GLA)	0.06	0.20^***^	0.12^**^	0.18^***^	0.15^**^	0.05^***^	0.03	0.13	0.06	0.16	0.12	0.03
Physical activity (IPAQ)	0.02	0.10^*^	0.09^*^	0.07	0.06	0.01	0.07	−0.01	0.07	0.06	0.03	0.01
Physical fitness (FFB-MOT)	0.00	0.12^**^	0.08	0.15^***^	0.01	0.03^**^	−0.12	0.06	−0.02	0.07	0.00	0.01
Strength	0.00	0.02	0.05	0.07	0.01	0.01	−0.08	0.02	−0.02	0.05	−0.03	0.01
CR-Fitness	0.01	0.10^*^	0.07	0.12^**^	0.00	0.02^*^	−0.17^*^	0.02	−0.09	0.04	−0.03	0.01
Flexibility	0.01	0.11^**^	0.07	0.15^***^	0.09^*^	0.02^*^	0.01	0.04	0.08	0.04	0.18^*^	0.03
Coordination	0.00	0.09^*^	0.03	0.10^*^	−0.05	0.02^*^	−0.09	0.07	−0.02	0.08	−0.13	0.03
Health behaviors (MHB-39)	−0.01	0.12^**^	0.03	0.17^***^	0.10^*^	0.03^**^	−0.01	−0.02	−0.16	−0.02	−0.03	0.03
Safety	0.05	0.00	0.02	0.08^*^	0.11^**^	0.02^*^	0.00	0.00	−0.08	−0.02	−0.05	0.00
AWOL	0.12^**^	0.33^***^	0.25^***^	0.24^***^	0.24^***^	0.14^***^	0.10	0.16^*^	0.04	0.09	0.17^*^	0.05
Compliance	−0.09^*^	−0.03	−0.12^**^	−0.03	−0.13^**^	0.02^*^	−0.10	−0.13	−0.13	−0.20^*^	−0.13	0.05
Diet	−0.01	0.06	0.04	0.11^**^	0.05	0.01	−0.02	0.04	0.06	0.06	0.06	0.01
Substance consumption	0.09^*^	0.09^*^	0.11^*^	0.05	0.04	0.02	−0.10	0.05	0.07	−0.13	0.08	0.06
Hygiene	−0.07	−0.02	−0.07	0.05	−0.06	0.02	−0.01	−0.09	−0.15	−0.01	0.03	0.03

N = 529 for self-ratings, N = 128-141 for peer-ratings. SMAP, short measure of adult playfulness; OTD, Other-directed; LTH, Lighthearted; INT, Intellectual; WHI, Whimsical playfulness.CR-Fitness, Cardio-Respiratory Fitness. AWOL = Leading an active way of life. R^2^ (OLIW) = Explained variance by all playfulness facets combined, over the influence of gender and age.

* p < 0.05.

** p < 0.01.

*** *p < 0.001. Two-tailed*.

**Table 3:** Partial correlations of self- and peer-rated playfulness with different peer-rated indicators of health, activity, and fitness, controlled for gender and age.

**Peer-Ratings**	**Self**						**PEER**					
	**SMAP**	**OTD**	**LTH**	**INT**	**WHI**	***R*^2^ (OLIW)**	**SMAP**	**OTD**	**LTH**	**INT**	**WHI**	***R*^2^ (OLIW)**
Global activity level (GLA)	−0.04	0.16	0.11	0.23^**^	0.10	0.05	0.12	0.26^**^	0.03	0.18^*^	0.23^**^	0.11^**^
Physical fitness (FFB-MOT)	−0.17^*^	0.05	0.05	0.06	−0.06	0.01	−0.03	0.11	−0.02	0.15	0.07	0.03
Strength	−0.21^*^	−0.05	−0.06	0.02	−0.11	0.02	−0.13	0.05	−0.05	0.14	−0.02	0.03
CR-fitness	−0.13	0.07	0.11	0.06	−0.04	0.02	−0.05	0.11	−0.02	0.15	0.06	0.03
Flexibility	−0.09	0.11	0.04	0.11	0.04	0.01	0.03	0.13	0.08	0.15	0.17^*^	0.04
Coordination	−0.15	−0.02	0.01	0.04	−0.12	0.02	0.00	0.02	−0.11	0.06	−0.02	0.02
Health behaviors (MHB-39)	0.05	0.11	−0.02	0.08	0.07	0.02	0.19^*^	0.28^**^	−0.11	0.31^***^	0.05	0.15^***^
Safety	0.11	−0.04	−0.01	0.08	0.05	0.02	0.18^*^	0.02	−0.03	0.22^*^	−0.02	0.06^*^
AWOL	0.15	0.37^***^	0.22^**^	0.23^**^	0.19^*^	0.14^***^	0.27^**^	0.44^***^	0.13	0.32^***^	0.26^**^	0.23^***^
Compliance	−0.06	−0.13	−0.14	−0.15^*^	−0.13	0.04	0.00	0.15	−0.13	0.08	−0.15	0.09^*^
Diet	−0.13	0.12	0.08	0.09	0.11	0.02	−0.12	0.03	0.02	−0.01	0.00	0.00
Substance consumption	−0.02	0.03	0.15	0.04	0.02	0.02	−0.06	0.13	0.15	−0.03	0.05	0.05
Hygiene	−0.10	−0.05	−0.09	−0.01	−0.06	0.01	−0.12	−0.16	−0.14	−0.11	0.06	0.04

N = 128-141 for peer-ratings. SMAP, short measure of adult playfulness; OTD, Other-directed; LTH, Lighthearted; INT, Intellectual; WHI, Whimsical playfulness.CR-Fitness, Cardio-Respiratory Fitness. AWOL = Leading an active way of life. R^2^ (OLIW) = Explained variance by all playfulness facets combined, over the influence of gender and age.

* *p < 0.05*.

** *p < 0.01*.

*** *p < 0.001.Two-tailed*.

#### Analysis of the self-ratings of playfulness

Global playfulness was widely unrelated to health, activity, and fitness but showed associations with health behaviors such as leading an active way of life or substance consumption (both positive), and compliance (negative) in self-ratings. *Other-directed* playfulness was positively associated with mental health, activity and all fitness measures (with the exception of strength) and showed positive relationships to health behaviors overall (especially, active way of life and substance consumption). *Lighthearted* playfulness positively related to mental and physical health and activity but was unrelated to physical fitness. It showed positive relationships to leading an active way of life and substance consumption, and negative relationships to compliance. *Intellectual* playfulness was positively associated with mental health, the global activity level, and all fitness measures (with the exception of strength), and was positively related to health behaviors overall, safety, leading an active way of life, and diet. Finally, *Whimsical* playfulness was unrelated to health, and fitness (with the exception of flexibility), but went along with higher levels of global activity. Further, it was related to health behaviors overall, safety, leading an active way of life, and diet (positive), and compliance (negative).

#### Analysis of the peer-ratings of playfulness

When analyzing the peer-ratings (see Table 2), we found generally small effect sizes (mean *r* = 0.02; range = [−0.20;0.18]) and in most cases correlation coefficients failed to reach statistical significance. For a better understanding of the relationships between self- and peer-rated playfulness and the tested measures, we computed vector correlations^2^ to estimate their overlap. While the self- and peer-correlations showed only a comparatively small overlap for global (*r* = 0.38) and *Intellectual* (*r* = 0.43) playfulness, there was a more substantial overlap for *Other-directed* and *Lighthearted* (*r* = 0.64) as well as *Whimsical* playfulness (*r* = 0.56). Hence, the inspection of single coefficients showed that differences (e.g., different signs for associations) in the correlational patterns existed particularly for global and *Intellectual* playfulness.

We analyzed the relationships between *peer-ratings* of health behaviors, activity, and fitness, and self- and peer-rated playfulness (see Table 3) and, again, computed the agreement of the relationships via vector correlations. When using the peer-rated perspective toward health behaviors their relationships with self- and peer-ratings of playfulness showed greater convergence, as it was substantial for global (*r* = 0.87), *Other-directed* (*r* = 0.76), *Lighthearted* (*r* = 0.85), and *Whimsical* (*r* = 0.73) while the lowest overlap existed for *Intellectual* playfulness (*r* = 0.51). Hence, the relationships between self- and peer-rated playfulness seem to widely converge in the sense that they share overlap in their relationships toward physical activities and health. However, the findings do not indicate a perfect overlap and should be interpreted cautiously. In any case they support the notion that the reported associations cannot be explained by a common method bias in the self-ratings of playfulness and the other measures.

Peer-rated global playfulness positively related to some health behaviors (mostly leading an active way of life) and showed some negative relationships to fitness. Most playfulness facets (*Other-directed, Intellectual*, and *Whimsical*) were positively related to activity, and went along with leading an active way of life.

Finally, we analyzed the relationship between self-rated health behavior and the aggregated self- and peer-ratings for playfulness to provide a more accurate estimate of the traits. As expected, the use of aggregated self- and peer-ratings contributed to the explanation of the relationships between playfulness and indicators of health (e.g., *R*^2^ increase ≤ 18%; see Supplementary Table C). The analyses of aggregated ratings widely confirmed the findings. Again, *Other-directed, Intellectual*, and *Whimsical* playfulness showed positive relationships to activity and leading an active way of life. However, while the consideration of peer-ratings might reduce the influence of biases due to the shared method, peer-ratings did not provide substantial additional information: They did not explain additional variance in health, activity, or fitness variables above the influence of self-ratings.

### Discussion

This study provides support for the notion of a contribution of playfulness to physical functioning. The findings were widely in line with expectations and show differential effects for the single facets of playfulness. As expected, self- and peer-ratings for playfulness and the indicators for health, activity, and fitness converged very well. As in previous studies (e.g., Proyer, 2017; Proyer and Brauer, 2018), playfulness and its facets were accurately perceived by their acquaintances. Thus, it can be concluded that peer-raters can observe playfulness and physical functioning well, and that their perception does not differ strongly from the self-perceptions by the individuals. These findings support the notion that the results obtained for self-reports are not an artifact due to the usage of the same method of assessment. This is also corroborated by the fact that most relationships of playfulness and health, activity, and fitness were somewhat parallel in self- and peer-reports (despite the expected lower coefficients in the analyses of the peer-ratings). However, vector correlation analyses indicated that relationships between self- and peer-ratings of *Intellectual* playfulness differed from each other independently of whether the outcome was assessed by self- or peer-ratings. In line with previous studies (e.g., Proyer and Brauer, 2018; Proyer et al., 2018a), we argue that the comparatively low observability of *Intellectual* playfulness contributes to differences in self- and peer-views and the associations toward external variables.

When combining the findings of the different data sources and focusing on those that were found in multiple combinations of self- and peer-ratings, *Other-directed* playfulness showed positive relationships with mental health, while no relationships with physical health were observed. The global level of activity (i.e., GLA) showed the most robust associations with *Other-directed* and *Intellectual* playfulness. For physical fitness, no robust relationships across multiple data sources were observed, except for a positive correlation between flexibility and *Whimsical* playfulness and small relationships between *Intellectual* playfulness and cardiorespiratory fitness and flexibility. Health behaviors overall were mostly positively related to *Other-directed* and *Intellectual* playfulness. All facets of playfulness were positively related to pursuing an active way of life; as expected *Other-directed* playfulness was the numerically strongest correlate in self- and peer-ratings. Finally, *Intellectual* playfulness was positively related to safety and negatively to compliance, whereas *Lighthearted* playfulness positively related to substance consumption.

Thus, based on the findings of Study 1, we conclude that there are positive relationships of playfulness with activity and mental health (for *Other-directed* and *Intellectual* playfulness). In line with the literature (e.g., Yang et al., 2016), one might argue that engaging in social acts contributes to engagement in health behavior. However, it should be tested whether being high in *Other-directed* playfulness is, indeed, correlated with engagement in group-sports (e.g., being in a soccer team). The relationships between *Intellectual* playfulness and activity/health might reflect previous findings of the correlation between cognitive ability and engaging in health behaviors (e.g., Gottfredson and Deary, 2004). Thus, it is not surprising that those preferring complexity over simplicity also show higher inclinations to lead an active life and report higher fitness. Overall, the associations of playfulness with physical fitness were positive and there was a robust relationship of all playfulness facets with leading an active way of life. However, there are also some aspects of playfulness that might also have negative consequences and go along with negative health behaviors (such as substance consumption or lacking compliance). Overall, the findings showed that playfulness is mostly positively associated with indicators of mental and physical health. Thus, one might argue, that those high in playfulness are at an advantage for health-related outcomes. However, causality cannot be determined, thus, it is unclear whether playfulness facilitates being active and striving for health or vice versa.

Study 1 has several limitations. The male:female ratio was imbalanced, as women were over-represented in the sample. While we control for the impact of age and gender in our analyses, it must be acknowledged that the study should be replicated using a more balanced sample. While we would argue that the inclusion of peer-ratings is a strength of the study this approach also has certain problems. For example, we did not control for the type of acquaintance (e.g., romantic partner, friend, family member, work colleague etc.; see Funder et al., 1995), which may have an effect on the findings. There is evidence that stronger acquaintanceship contributes to the accuracy of perceiving others' personality (e.g., Watson et al., 2000) and one might expect that accuracy for judging health behaviors would also be highest in highly acquainted peers. Taking into account that all peer-raters were at least friends with whom they rated and that there was substantial self-peer convergence for all measures, one might argue that holding acquaintanceship constantly high (e.g., by exclusively employing romantic partners as peer-raters) would be the best option to gather an accurate estimate of health behaviors. On a more global level, the inclusion of the peer-ratings show that the contribution of playfulness to the understanding of the different activity-measures do not seem to be based on a joint method-bias (i.e., all self-ratings), but have substance above and beyond similarities in the way the data were collected. Another limitation concerns the assessment of physical and mental health, as these were assessed with 1-item measures. Although these correlated with measures of fitness, activity, and health behaviors single-item reliability cannot be estimated and thus warrants cautious interpretation.

Although in our sample healthy and active people were slightly overrepresented (slightly negative skewed distributions for health-related variables), the sample covered a broad range from very active and healthy to not very active and unhealthy people. The same was true for playfulness: The whole range of the theoretical scale in the playfulness measures was represented and means were comparable to what has been reported before for samples from the general population (Proyer, 2012a, 2014b).

## Study 2

In Study 2, we address some limitations of Study 1 by testing the association of playfulness with physical activity and fitness with different, more rigorous methodological approaches, including objective measures. We used an interview approach for assessing physical activity. The strength of this approach is that we had the possibility of directly inquiring about activity and discussing questions with each of the participants, rather than have to rely on the answers in questionnaires. Of course, it must be acknowledged that these data are also self-reports. Additionally, we administered a broad array of field tests, covering cardiorespiratory fitness (climbing stairs; Boreham et al., 2000), flexibility (stretching; Wells and Dillon, 1952), strength (hand-grip strength; ACSM, 2001), endurance (repeatedly standing up from a chair; Bohannon, 1995), and dexterity (placing pins with both hands simultaneously in small holes on a metal plate; Schoppe, 1974) for objective assessments of physical fitness. These tasks were selected to represent a broad range of indicators of fitness, which allows differentiating among these components. Hence, Study 2's main contribution is the inclusion of objectively measured indicators of fitness.

As in Study 1, playfulness was assessed with established self-report measures. However, we also included indicators of playful behaviors directly. Two types of behaviors were considered. Firstly, we observed participants during a waiting period and assessed how many playful items they interacted with deliberately during this period. The items were pre-defined (see procedure for details). The expectation was that greater levels of playfulness would be associated with more playful activities in a standardized time period. Secondly, participants completed the task for assessing their dexterity twice; namely, once under the standard condition and once in an impaired condition using goggles that simulate different levels of alcohol intoxication. These are typically used within driver education programs to demonstrate varying degrees of visual impairments due to intoxication. Participants could freely choose the level of impairment for doing this task and were allowed to try them out as long as they wanted. We expected that playful people select stronger levels of impairment as this would allow for greater expressions of their playfulness and since playfulness is associated with a mastery orientation (Proyer, 2014a) and a liking of competitiveness (Csikszentmihalyi, 1975). Finally, we assessed body height and weight to control for influences of participants' body mass index in addition to controlling for gender and age as in Study 1.

Based on the results of Study 1, we expected positive associations of *Other-directed* and *Intellectual* playfulness with all tested aspects of activity and fitness. For dexterity, we expected even stronger relationships in the impairment condition than in the standard condition, since playful people are hypothesized to adapt quicker to new circumstances and are willing to work under less structured external conditions (e.g., Proyer, 2012a, 2014a). Finally, we expected these associations with fitness also to be present in the more objective indicators of playfulness.

### Methods

#### Sample

A total of *N* = 67 participants (73.1% women) aged from 19 to 75 (*M* = 39.21, *SD* = 18.54) took part in Study 2. A large part of the sample (43.3%) has a degree from a university or a university of applied sciences, 40.3% have a degree allowing them to attend a university or a university of applied sciences, 14.9% completed vocational training, and 1.5% have completed mandatory school. Most participants (89.6%) are Swiss. About half of the participants were currently in a relationship (70.1%), 26.9% single, and 3.0% were divorced or separated. Most participants were employed (61.2%). Thus, based on the demographic composition, the sample is highly comparable to the sample of Study 1. Further, as planned, we have successfully over-sampled low-scorers (scores of 3 and below; 20.9%) and high-scorers (scores of 5 and above; 41.3%) for being able to differentiate among participants with more extreme expressions, rather than having a larger number of participants in the middle range. Finally, the sample was also diverse with regard to their body mass index, which ranged from 16.5 to 31.0 (*M* = 22.40, *SD* = 3.23).

A power analysis showed that the study's sample size allowed detection of correlation effects of ρ = 0.28 with a power of 0.80 (α = 0.05). Thus, medium-to-large effects can be detected through conventional null-hypothesis significance testing. Therefore, we will interpret correlations in terms of its effect size. Our effect size of interest is set at *r* = 0.21 following Gignac and Szodorai's (2016) recommendations for studies on individual differences.

#### Instruments

##### Questionnaire measures

As in Study 1, the *Short Measure of Adult Playfulness* (SMAP; Proyer, 2012a) and the OLIW-playfulness questionnaire (OLIW; Proyer, 2017) were used. Internal consistencies were acceptable (SMAP: α = 0.90; OTD: α = 0.74, LIG: α = 0.68, INT: α = 0.68, WHI: α = 0.83).

The *International Physical Activity Interview* short-form (IPAQ; Craig et al., 2003) asks about the time spent with physical activity in the past 7 days. The time spent with physical activity of low, moderate, and vigorous levels of intensity is assessed, and, additionally, the time spent sitting. Craig et al. (2003) report acceptable measurement properties.

##### Objective measures

We assessed participants' body height and weight using standard instruments in order to control for the influence of body mass index in subsequent analyses.

The *Hand-Grip Strength Test* (ACSM, 2001) is an indicator of isometric strength of the hand and forearm muscles. This is measured using a hand dynamometer that has to be squeezed with the dominant hand as hard as possible. The standard instruction allows the participants to try three times in a row while only the best trial is recorded. Bohannon (1998) reports high convergent validity with other measures of arm strength. In the present study, a steel spring dynamometer (Collin's) was used.

The *1-min Sit-to-Stand Test* (Bohannon, 1995) is a measure for lower body muscular strength and endurance (representative normative data for Switzerland have been published by Strassmann et al., 2013). Participants are instructed to stand up and sit back down on a chair as many times as possible during 1 min. Ritchie et al. (2005) report good reliability and convergent validity with other measurements.

The *Stair-Climbing Exercise* (STE; Boreham et al., 2000) is an indicator for overall cardio-respiratory fitness. Participants walked 100 steps (i.e., 10 flights of 10 stairs) at a fixed, metronome-paced speed (i.e., 90 steps per minute). The variable of interest was the change in heart rate. Heart rate (beats per minute; bpm) was monitored continuously during the exercise, and during short periods before (5 min) and after the exercise (1 min), for having estimates for baseline heart rate, and the recovery of the heart rate after exercise. Heart rate was measured using a commercial device (Polar H7) using a chest strap that has been shown to yield highly reliable results that are comparable to electrocardiogram measurement (Wang et al., 2017).

The *Sit and Reach test* (Wells and Dillon, 1952) is a test of back and leg flexibility. Participants sit on the floor and bend their arms forward, as far as possible. The standard instruction allows the participants to try two times in a row while only the best trial is recorded. Wells and Dillon (1952) report good reliability and validity of this task, and others report good criterion validity for hamstring extensibility (Mayorga-Vega et al., 2014). In the present study, the “zero point,” where the participant's fingertips reach as far as their feet, was set at 26 cm.

The *Test of Fine Motor Functions* (TOFMF; Schoppe, 1974) is a test for hand- and finger dexterity. The participant has to take short pins out of a box and put them as quickly as possible in the corresponding hole, with both hands simultaneously. We measured the time between the first pin is set and the last pin is set. Hamster (1980) reports good convergent validity of this task with other motor tasks. This test was conducted twice; once under standard conditions, and once under an impaired vision condition, using “Drunk buster goggles” that simulate reduced alertness, slowed reaction time, confusion, visual distortion, alteration of depth and distance perception, reduction of peripheral vision, poor judgment and decision making, and lack of muscular coordination.

#### Procedure

As in Study 1, we recruited participants through online-advertisements (forums, mailing lists), and by contacting participants from earlier (unrelated) studies. The participants completed online versions of the playfulness instruments. After completion, they were invited to the lab study that took place at the University of Zurich. Depending on the availability of the participants, the lab study took place a couple of days to several weeks after the completion of the online study. Three instructors (two psychology students and one graduate student) were trained by the principal investigators to conduct the lab studies. This study had two parts, an activity part and a playfulness part. At the beginning of the lab study, participants were informed again on the study and gave informed consent. Afterwards, the participants' height and weight were measured and participants put on the chest strap for measuring the heart rate. Then, the interview on the physical activity during the last 7 days (IPAQ) was conducted. The administration of the IPAQ took about 5 min on average. We measured the participants' heart rate during 5 min while sitting. Afterwards, participants conducted the Stair-Climbing Exercise. During the stair climbing, the heart rate was continuously measured. Immediately after the stair climbing, participants sat down for 1 min during which the recovery of the heart rate was measured. Afterwards, participants completed the Hand-Grip Strengths Test, the Sit-To-Stand Test, and the Sit-And-Reach Test.

Subsequently, participants were told that the instructor has to process the so far collected data and they have to wait for about 5 min. However, they were invited to look at some materials that will be used later on in the experiment (six in total). Participants were introduced to three “drunk buster goggles” that simulate different degrees of alcohol intoxication (i.e., 0.04–0.06%, 0.06–0.08%, and 0.08–0.15% blood alcohol content [BAC]). The participants were invited to try out and play with some items (e.g., a yo-yo, a Rubik's cube, a marble labyrinth). During these 5 min, the instructor was paying attention to which goggles the participants try on and what items they played with, for assessing objective indicators of playful behaviors. Afterwards, participants conducted the Test of Fine Motor Functions twice: Once in a normal, unimpaired condition and a second time using a drunk buster goggle of their choice. Together with the participants' behaviors during the waiting time, this choice should serve as a more objective indicator for playfulness. Finally, the participants were debriefed and received a reimbursement of 25 Swiss Francs for their participation. The full procedure of Study 2 is given in Table 4.

**Table 4:** Schematic of study 2.

Online	Self-report measures on playfulness (SMAP, OLIW)
On Site	Introduction; informed consent
	Assessment of body height and weight
	Interview on physical activity (IPAQ)
	*Activity Part*
	•Cardiorespiratory measure (Stair-Climbing Exercise; including Baseline and Recovery assessment of heart rate)
	•Hand-Grip Strength Test
	•Sit-To-Stand Test
	•Sit-And-Reach Test
	*Playfulness Part*
	•Waiting period (5 min)
	•Test of Fine Motor functions (unimpaired)
	•Test of Fine Motor functions (impaired)
	*Debriefing*

### Results

First, we analyzed the relationships of playfulness and its facets with the data from the interview on physical activity in the last 7 days, in order to replicate the findings of Study 1 using a different methodology (Table 5) and while also controlling for gender and age. Additionally, we have controlled for body mass index due to its relationship to both playfulness (mostly *Lighthearted* playfulness [*r* = 0.27, *p* = 0.028] but also nonsignificant trends for *Intellectual* [*r* = 0.14], *Whimsical* [*r* = 0.13], and *Other-directed* [*r* = 0.08] playfulness) and fitness measures (mostly recovery heart rate [*r* = 0.35] and lower body strength and endurance, *r* = −0.40).

**Table 5:** Partial correlations of playfulness (SMAP) and its facets (OLIW) with self-reported activity in the last 7 days.

	**SMAP**	**OTD**	**LTH**	**INT**	**WHI**	**Items**	**Goggle**
Activity total	0.13	0.29^*^	0.26^*^	0.18	0.14	0.21^*^	0.05
Time spent walking	−0.01	0.08	0.25^*^	0.17	−0.15	−0.12	0.17
Time spent with moderate activity	0.14	0.24^*^	0.06	0.19	0.11	0.18	0.17
Time spent with vigorous activity	0.13	0.22^*^	0.12	0.02	0.26^*^	0.30^**^	−0.15
Time spent sitting	−0.33^**^	−0.25^*^	−0.21^*^	−0.15	−0.06	−0.01	−0.05

Note N = 67. All correlations are controlled for gender, age, and body mass index.SMAP, short measure of adult playfulness; OTD, Other-directed; LTH, Lighthearted; INT, Intellectual; WHI, Whimsical playfulness. Items = Number of items played with (range = 0–6); Goggle = Strength of chosen goggle (range = 0.04–0.06% blood alcohol content (BAC) = 1;0.06-0.08% BAC = 2;0.08-0.15% BAC= 3).

* *p < 0.05*.

** *p < 0.01. One-tailed*.

Table 5 shows that *Other-directed* and *Lighthearted* playfulness were positively related to the total amount of time spent physically active in the last 7 days as derived from the interview, and negatively related to the amount of time spent sitting (as did global playfulness). *Other-directed* playfulness went along with more time spent with moderate or vigorous activity, whereas *Lighthearted* playfulness was positively related to the time spent walking. The other playfulness facets and global playfulness showed fewer and smaller relationships to the different types of activities. When looking at the more objective indicators of playfulness, we found positive associations between the number of items participants played with and total activity and the time spent with vigorous activity. Further, the level of impairment of the chosen goggle showed a tendency to go along with more time spent walking and with moderate activity.

Second, we examine the objective measures of fitness and strengths and their relationships with playfulness and its facets (Table 6).

**Table 6:** Partial correlations of playfulness and its facets with objective measures of fitness, strength, and dexterity.

				**Correlations**						
	***M***	***SD***	**Range**	**SMAP**	**OTD**	**LTH**	**INT**	**WHI**	**Items**	**Goggle**
Baseline heart rate (bpm)	780.60	100.54	56–109	−0.19	−0.26^*^	−0.10	−0.18	−0.07	−0.01	−0.18
Activity Heart Rate (bpm)	1190.14	140.25	88–151	−0.24^*^	−0.32^**^	−0.15	−0.25^*^	0.01	0.02	−0.07
Recovery Heart Rate (bpm)	980.21	200.58	59–146	−0.23^*^	−0.27^*^	−0.10	−0.14	−0.22^*^	−0.11	−0.14
Hand and Forearm Strength	650.92	210.69	27–127	0.13	0.30^*^	0.23^*^	0.61^***^	0.22^*^	−0.02	0.15
Back and Leg Flexibility (cm)	310.57	90.48	5–46	0.10	0.02	0.27^*^	0.12	0.24^*^	0.13	0.04
Lower Body Strength and Endurance (number of repetitions)	350.03	90.18	18–61	0.06	0.20	0.07	−0.11	−0.06	0.24^*^	0.09
Hand and Finger dexterity, standard condition (s)	770.15	200.89	50–173	−0.15	−0.20	−0.01	0.01	−0.04	0.04	−0.11
Hand and Finger dexterity, impairment condition (s)	1330.01	640.98	70–520	−0.02	0.04	−0.17	0.18	−0.20	–	–

N = 61-67. All correlations are controlled for gender, age, and body mass index Correlations with fine motor skills in the impairment condition are controlled for the selected goggles SMAP, Short Measure of Adult Playfulness; OTD, Other-directed; LTH, Lighthearted; INT, Intellectual; WHI, Whimsical playfulness Baseline Heart Rate = Average heart rate (bpm) while sitting for 5 min; Activity Heart Rate = Average heart rate (bpm) during stair climbing exercise; Recovery Heart Rate = Average heart rate (bpm) during 1 min after the exercise Hand and Forearm Strength = Arbitrary scale; Back and Leg Flexibility = Distance participants were able to bend their arms forward (“zero point” = 26 cm); Lower Body Strength and Endurance = Number of repetitions in the 1-min Sit-to-Stand Exercise; Hand and Finger dexterity = Seconds to complete the Test of Fine Motor Functions in standard condition or impairment condition (using a goggle simulating alcohol intoxication) Items = Number of items played with (range = 0 to 6); Goggle = Strength of chosen goggle (range = 0.04-0.06% blood alcohol content (BAC) = 1;0.06-0.08% BAC = 2;0.08-0.15% BAC = 3).

* p < 0.05.

** p < 0.01.

*** *p < 0.001. One-tailed tests*.

The table shows that playfulness demonstrated mostly the expected pattern with objective of fitness, strength, and dexterity: Lower heart rates (i.e., average heart rate at baseline, during the stair climbing, and during recovery) went along with higher scores in global playfulness, *Other-directed* playfulness, while there were also some effects for *Intellectual* and *Whimsical* playfulness. All playfulness facets—but not global playfulness—were positively related to hand and forearm strength. *Lighthearted* and *Whimsical* playfulness went along with better back- and leg flexibility. *Other-directed* playfulness tended to go along with better lower body strength and endurance. Also, there were trends toward positive relations to hand and finger dexterity in the unimpaired condition, while *Lighthearted* and *Whimsical* playfulness went along with better performance and *Intellectual* playfulness with worse performance in the impairment condition.

Finally, we analyzed the relationships of the objective indicators of playfulness; these are the number of items the participants played with during the waiting time (ranging from 1 to 6; *M* = 4.69, *SD* = 1.36) and the strength of the chosen impairment in the goggle that is, the level of simulated alcohol intoxication. Most participants (59.7%) chose the weakest goggle simulating a blood alcohol content (BAC) of 0.04–0.06%, 16.4% chose the goggle of medium intensity (0.06–0.08% BAC), and 23.9% chose the strongest goggle (0.08–0.15% BAC). Preliminary analyses showed, that these indicators were indeed related to playfulness: The strength of the chosen goggle positively related to global (*r*[62] = 0.32, *p* = 0.005) and *Lighthearted* playfulness (*r*[62] = 0.21, *p* = 0.048), whereas the relationships with the other facets were mostly in the intended direction, but did not reach significance (*Other-directed*: *r*[62] = 0.17, *p* = 0.089; *Intellectual*: *r*[62] = 0.19, *p* = 0.067; *Whimsical*: *r*[62] = 0.01, *p* = 0.480). The number of items played with was unrelated with self-rated playfulness (all *r*s < 0.09), and the strength of the chosen goggle (*r* = 0.09, *p* = 0.511). Since these indicators do not allow for fine-grained distinctions and are rather rough assessments of playful behavior, we considered these associations with the trait-measures of playfulness to be adequate for considering these indicators as objective measures of playfulness.

Further, we found that the number of items participants played with during the waiting time was associated with greater strength and endurance in the lower body. Also, those who chose goggles simulating higher levels of intoxication tended to show a lower baseline heart rate. No other robust effects were found (|*r*| < 0.16, *p*s > 0.10) but with few exceptions, all effects were in the expected direction and were highly similar to those from the self-reports on playfulness.

### Discussion and general discussion

Study 2's main contribution is corroborating findings from Study 1 with multiple methods and adds objective measures of physical activity to research in adult playfulness. The findings widely confirmed the results of Study 1 regarding activity with the strongest relationship found for *Other-directed*, and tendencies for *Intellectual* playfulness. In this study we also found evidence in favor of *Lighthearted* playfulness. Further, objective indicators of playfulness also yielded some positive associations with activity. In contrast to Study 1, we also detected relationships between playfulness and fitness: global, *Other-directed*, and *Intellectual* playfulness positively related to cardiorespiratory fitness, while all playfulness facets positively related to measures of strength. For dexterity, only small effects in the expected direction were obtained. Also, the objective indicators of playfulness only yielded few robust associations, but were generally in the expected direction.

The more exploratory analyses on the self-selected visual impairment show that greater playfulness was associated with the selection of greater impairments, which may increase the play-experience, but also could be interpreted as a sign of competitiveness. The latter has already been discussed in previous research in its association with play and playfulness (e.g., Rogers et al., 1987; see also Csikszentmihalyi, 1975). It needs mentioning that the selection of the degree of visual impairment is only one potential behavioral indicator of playfulness. This is a comparatively new line of research (linking trait playfulness in adults with *miniature situations* representative of the actual behavior associated with the trait) and warrants further verification. As noted for so-called objective personality tests in the Cattellian tradition (allowing for the assessment of *T*-data; for an overview see Ortner and Proyer, 2018) single tests assessing a specific behavior do not correlate systematically with self-reports (as in our study). Hence, one aim of future research will be the development of further objective tests for the assessment of playfulness and aggregate them for validation studies (e.g., when relating them to self-reports) and their validation against other data sources (e.g., *L*-data). Nevertheless, a limitation for the usage of the selection of the degree of visual impairment as an approximation for playful behavior is that it will require further validation in future studies.

Again, this study highlights the importance of differentiating among different facets of playfulness as they have different predictive value. The findings also show that a global assessment can only give a general sense of the direction of the associations, but cannot provide a more fine-grained differentiation. It should be noted that the size of the correlation coefficients between *Hand and Forearm Strength* and *Intellectual* playfulness (about 37% shared variance) is an anomaly in comparison with the other coefficients. This finding requires replication (as is warranted for the other findings) and most likely seems to be attributable to specifics of this particular sample. While we caution against over-interpretation, future research is warranted to test how robust this association is.

Several limitations in these studies must be addressed: Again, the sample is imbalanced with respect to the male:female ratio. Further, the question of the generalizability of the findings needs to be discussed. We have tested a rather diverse sample in Study 1 (also in Study 2, but of smaller size), but, of course, the samples are not fully representative, nor gender balanced. However, since most relationships with demographic characteristics were rather small, and we corrected for influences of age and gender, we do not have much reason to doubt the validity of the findings. However, it is possible that there was a sampling effect (i.e., studies on activity mainly attract active individuals). Although our sample was diverse in this regard, we did not find participants that were not physically active at all. Thus, some effects might be underestimated in the present study since no physically non-active participants were tested. While we were able to replicate some findings of Study 1 with Study 2, some reported effects (those on the more objective measures) warrant replication in future studies using independently collected and more representative samples. Although coefficients were in the expected range, the sample size allowed us to detect mainly medium-to-large effects through testing statistical significance. Hence, we interpreted smaller effects cautiously and upon effect size (cf. Gignac and Szodorai, 2016) instead of relying solely on statistical significance. However, future studies require larger sample sizes to detect potential small effects. Whereas the findings show that playfulness is related to health, activity, and fitness, it also became apparent, that the relationships are of small-to-medium size. Replication and extension is warranted.

Overall, our studies show that playfulness is positively correlated with greater levels of activity, specific aspects of fitness (mostly cardiorespiratory fitness), and specific health behaviors (mostly leading an active way of life). Whereas the different facets of playfulness vary in their relationships with indicators of physical activity, *Other-directed* playfulness seems to be the most relevant aspect to levels of fitness, activity, and health, as it yielded the strongest and most robust associations across studies and methods, followed by *Intellectual* and *Lighthearted* playfulness. A future research direction could be disentangling the effects of social activities and a social inclination in general and testing the specific contribution of *Other-directed* playfulness (e.g., being able to interact playfully with others to have more fun during the exercises, or using joint activities as an additional resource to promote fitness). *Whimsical* playfulness, on the other hand, seems to be widely unrelated to physical activity (with exceptions, though—e.g., it was associated with more reported time spent with vigorous activity in Study 2). Hence, future studies should examine the distinct effects of single facets in the prediction of health-related outcomes.

The relationships reported in these studies are generally small in terms of size but robust across different methods and cannot be explained by a method bias (shared method variance). The relationships can also be found when using more objective measures of physical activity, rather than the self- and peer-ratings. As expected, correlations are smaller due to the use of different methods. However, most relationships between playfulness and physical activity were in the expected direction.

This research was conducted cross-sectionally therefore, does not allow for the interpretation of causality (or the direction of the relationships between playfulness, and health, activity, and fitness). However, an initial working model could be proposed to conduct research that explores the direction of the relationship and test underlying mechanisms between playfulness and physical activity, and mental and physical health. Such a model could be framed in the context of health behavior models. Personality variables can affect health through influencing a person's compliance with health-oriented behaviors (e.g., Wiebe and Smith, 1997; Vollrath et al., 1999; Kubzansky et al., 2009) and between personality and exercise behavior (e.g., Rhodes and Smith, 2006; Allen et al., 2017). However, none of these have looked at the personality trait of playfulness. One of the most influential models to be applied to physical activity is the *Theory of Planned Behavior* (TPB), a social-cognitive model that proposes attitudes and beliefs (e.g., perceived ability to be active, and perceptions about what other people think about it), influence intentions to be active, which in turn determines actual behavior (e.g., Ajzen, 1985). Individual factors such as personality are thought to differentially influence the role of these predictors in the model (see Ajzen, 2011) Indeed, research suggests that some personality factors may play a role in moderating determinants within the TPB upon physical activity (e.g., Courneya et al., 1999; Rhodes et al., 2005; Vo and Bogg, 2015), although to the best of our knowledge, playfulness has not yet been investigated. It is plausible that playfulness could exert effects upon motivational and volitional aspects of physical activity goal pursuit. For example, playful people that enjoy group exercise and socializing, or reframe activity to make it more entertaining, may be more likely to enjoy physical activity, and have routines that promote regular exercise adherence. Currently, we also expect that there are many, bi-directional links between playfulness and physical activity and overall well-being. Furthermore, these links likely operate directly and/or be mediated by current well-being/health.

As discussed earlier, there is evidence for a positive relationship between being playful and the experience of positive emotions (e.g., joy or contentment). Positive affect may have a wide ranging influence on well-being—very much in the sense of Fredrickson's (2001) notion of a positive upward spiral associated with the experience of positive emotions (see also Panksepp, 1993). This relationship may also be helpful for a better understanding of why playfulness relates to physical activity or leading an active way of life in general. More specifically, positive affect could influence physical activity goal pursuit (e.g., Cameron et al., 2015, 2017) for example via the feelings-as-information route, whereby people interpret how they feel in general to be a favorable judgment about a target health behavior. Future tests can clarify the impact of positive affective states upon physical activity levels.

Another potential pathway that warrants further investigation is the idea that playfulness improves flexible thinking (i.e., the ability to shift perspectives, seeing new solutions, and adapting to new situations). *Intellectual* types of playfulness (Proyer, 2017) may be particularly useful for the pursuit of, and engagement in physical activities (e.g., in the sense of generating interest, or for maintaining high motivation). More broadly, psychological flexibility may be a fundamental aspect of psychological health (Kashdan and Rottenberg, 2010) and may have the potential of also contributing to physical well-being and activity.

Future studies should examine these hypotheses in both acute studies and within longitudinal designs to assess potential health-benefits and outcomes (e.g., longevity) in more detail. If playing and being playful facilitates the emergence of positive emotions (e.g., Fredrickson, 2001) and, amongst others, contribute to better coping with stressors (e.g., Staempfli, 2007; Magnuson and Barnett, 2013; Proyer, 2014a), there may also be long-term effects observable (see Gordon, 2014). Finally, it should be acknowledged that in biology there is the idea that animals primarily play when they feel safe and have enough energy to do so (e.g., when being healthy; for an overview see Burghardt, 2005). Hence, there may be a different working mechanism to consider: Only those that are healthy and not exposed to severe psychological or environmental stressors and active can “afford” to play, while others must be more protective of their available resources.

The next logical step from our perspective would be devising intervention studies. For example, measuring playfulness (or specific facets) and testing whether this influences physical activity determinants and levels over time. Experience sampling methods, where participants indicate levels of playfulness, positive affect and activity across the day, could elucidate the interplay between these components, and help identify relevant situations and behaviors for intervention studies. Another area to research would be the fit between type of physical activities pursued and preference in specific domains of playfulness. In this respect, observation studies of extreme groups in natural environments would be helpful (e.g., observing sport teams, or other people pursuing different types of physical activities) to relate behavior to playfulness. Appealing to an adult's sense of playfulness seems advisable when developing interventions to increase engagement in physical activity. One might think of the development of a program that facilitates physical activity in a way that demands certain levels of playfulness (e.g., by embedding competitions, facilitating playful interactions with others, playing for a “reward,” or having playful reinforcers such as those involving humorous content). This trend has already started and various behavior change programs have been developed with the goal of “gamifiying” more traditional interventions (e.g., Howells et al., 2016), and has also been suggested for interventions aiming at addressing health (Cugelman, 2013).

A caveat of our research is that we have not covered potential negative effects of play and playfulness. For example, Burghardt (2005) lists examples of cruel aspects of play (e.g., when cats play with their prey, killing it slowly) as well as its risky, dangerous, or addictive components (e.g., when playing risky types of sports and similar behavior)—hence, play and playfulness may not always be fun nor positively contributing to health and well-being (e.g., when experimenting with substances). Further, we did not assess variables such as consumption of legal and illegal drugs, medical conditions, or physical restrictions—these variables might also affect the relationships between playfulness, activity, and health, or limit the relationships that can be found in such a study (a playful individual could be more active if s/he would not suffer from a medical condition). Therefore, it may be necessary to have an even more fine-grained list of actual play-behaviors (e.g., Proyer, 2017) and test which of them predicts health-behaviors and activity. The latter may also be helpful to address the question of whether playful people engage (exceedingly) in computer/online-based games and games played on mobile devices and whether this may also have detrimental effects on physical activity levels.

Finally, it would be interesting to extend the research on playfulness to other physical and mental skills; it might be the case that highly skilled individuals in a broad array of fields (e.g., arts, sports, or intellectual domains) might be higher in playfulness than the general population since learning and practicing skills might be facilitated by playfulness. In fact, Study 2 provided some first insights on how playful individuals might acquire skills: Those high in playfulness chose to perform the fine-motor task in a more playful way in the self-selected vision impairment condition–even if there was no necessity to do so and this leads to a decreased performance in a current task. However, this attitude of seeking and playing with challenge might explain how playfulness adds to the acquisition and mastering of new skills.

## Ethics statement

This study was carried out in accordance with the recommendations of the Swiss Psychological Association with written informed consent from all subjects. All subjects gave written informed consent in accordance with the Declaration of Helsinki. According to the local ethics committee (Kantonale Ethikkommission Zürich), the present study did not require a formal approval.

## Author contributions

RP, FG, and EB: conception and design of the work; RP and FG: data collection; RP, FG, EB, and KB: data analysis and interpretation; RP and FG: drafting the article; RP, FG, EB, and KB: critical revision of the article; RP, FG, EB, and KB: final approval of the published version.

### Conflict of interest statement

The authors declare that this study received funding from Unilever. EB was a staff member of Unilever R&D and contributed to the objectives, design of the study and the final manuscript.

## Footnotes

^1^Fredrickson (2003) notes: “For example, joy and playfulness build a variety of resources. Consider children at play in the schoolyard or adults enjoying a game of basketball in the gym. Although their immediate motivations may be simply hedonistic—to enjoy the moment—they are at the same time building physical, intellectual, psychological and social resources. The physical activity leads to long-term improvements in health, the game-playing strategies develop problem-solving skills, and the camaraderie strengthens social bonds that may provide crucial support at some time in the future […]. Similar links between playfulness and later gains in physical, social and intellectual resources are also evident in nonhuman animals, such as monkeys, rats and squirrels” (p. 333).

^2^Vector correlations were computed on basis of Fisher's *r*-to-*z* correlations (see Borkenau and Liebler, 1992).
