# Supplementary material for: The Positive Relationships of Playfulness With Indicators of Health, Activity, and Physical Fitness
Source: Front Psychol. 2018 Aug 14;9:1440. doi: 10.3389/fpsyg.2018.01440 (PMC6102740; doi:10.3389/fpsyg.2018.01440)
Supplement: Supplementary file 1 [file Table_1.DOCX]

Supplementary Material

The Positive Relationships of Playfulness with Indicators of Health, Activity, and Physical Fitness

René T. Proyer^12^, Fabian Gander^2*^, Emma J. Bertenshaw^3^, and Kay Brauer^1^

^1^ Personality and Assessment, Department of Psychology, Martin-Luther University of Halle-Wittenberg, Halle, Germany

^2^ Personality and Assessment, Department of Psychology, University of Zurich, Zurich, Switzerland

^3^ Unilever R&D, London, United Kingdom

*** Correspondence:**René T. Proyer
rene.proyer@psych.uni-halle.de

Supplementary Table A

*Convergence of Self- and Peer-Ratings of Playfulness and its Facets*

|  | Peer-Ratings | | | | |
| --- | --- | --- | --- | --- | --- |
|  | SMAP  (Global) | Other-directed | Lighthearted | Intellectual | Whimsical |
| Self-Ratings |  |  |  |  |  |
| SMAP (Global) | **.51** | .37 | .18 | .18 | .09 |
| Other-directed | .41 | **.47** | .19 | .23 | .14 |
| Lighthearted | .19 | .24 | **.52** | .34 | .30 |
| Intellectual | .20 | .08 | .23 | **.46** | .22 |
| Whimsical | .20 | .03 | .13 | .20 | **.55** |

*Note*. *N* = 141. SMAP = short measure of adult playfulness.

For all *r* > .17, *p <* .05; all *r* > .23, *p <* .01; all *r* > .29, *p <* .001. Two-tailed.

Supplementary Table B

*Convergence of Self- and Peer-Ratings of Health Behaviors, Activity, and Fitness*

|  | Peer-Ratings | | | | | | | | | | | | |
| --- | --- | --- | --- | --- | --- | --- | --- | --- | --- | --- | --- | --- | --- |
| Self-Ratings | (1) | (2) | (3) | (4) | (5) | (6) | (7) | (8) | (9) | (10) | (11) | (12) | (13) |
| 1. Global activity level (GLA) | **.64** | .37 | .32 | .36 | .23 | .24 | .32 | .08 | .32 | -.02 | .37 | .16 | .24 |
| 1. Physical Fitness (FFB-MOT) | .35 | **.74** | .57 | .67 | .48 | .67 | .08 | -.07 | .14 | -.08 | .22 | .10 | .12 |
| 1. Strength | .20 | .55 | **.63** | .57 | .20 | .45 | -.05 | -.12 | .02 | -.10 | .19 | .05 | .01 |
| 1. CR-Fitness | .37 | .67 | .58 | **.73** | .28 | .57 | .00 | -.14 | .11 | -.12 | .22 | .10 | .14 |
| 1. Flexibility | .19 | .31 | .08 | .08 | **.53** | .27 | .29 | .19 | .18 | .01 | .18 | .13 | .22 |
| 1. Coordination | .20 | .57 | .42 | .52 | .30 | **.59** | -.07 | -.16 | .04 | -.03 | .04 | -.03 | -.10 |
| 1. Health Behaviors (MHB-39) | .29 | .10 | .04 | .01 | .21 | .03 | **.56** | .26 | .20 | .22 | .35 | -.11 | .28 |
| 1. Safety | .02 | -.07 | -.11 | -.14 | .09 | -.09 | .33 | **.50** | -.01 | .04 | .08 | .04 | .04 |
| 1. AWOL | .19 | .22 | .13 | .16 | .26 | .16 | .08 | -.21 | **.47** | -.06 | .06 | -.03 | -.07 |
| 1. Compliance | -.09 | -.01 | -.07 | -.03 | .05 | .01 | .21 | -.03 | -.15 | **.47** | .14 | .02 | .11 |
| 1. Diet | .39 | .06 | .07 | .10 | .02 | -.01 | .28 | .12 | .17 | -.04 | **.55** | .27 | .21 |
| 1. Substance Consumption | -.05 | -.02 | -.09 | .08 | -.03 | -.06 | -.18 | -.07 | -.04 | -.03 | .02 | **.56** | .14 |
| 1. Hygiene | .24 | .03 | .08 | .01 | .06 | -.03 | .31 | .15 | .02 | .03 | .22 | .11 | **.68** |

*Note*. *N* = 128-141. For all *r* > .17, *p <* .05; all *r* > .23, *p <* .01; all *r* > .29, *p <* .001. Two-tailed.

Supplementary Table C

*Relationships of Averaged Self- and Peer-Ratings of Playfulness with Averaged Indicators of Health, Activity, and Fitness, Controlled for Sex and Age*

|  | SMAP | OTD | LTH | INT | WHI | *R*^2^ (OLIW) |
| --- | --- | --- | --- | --- | --- | --- |
| Global activity level (GLA) | .04 | .27** | .11 | .23** | .18* | .11*** |
| Physical Fitness (FFB-MOT) | -.15 | .09 | .02 | .13 | .01 | .03 |
| Strength | -.18* | .00 | -.04 | .07 | -.06 | .02 |
| CR-Fitness | -.15 | .09 | .03 | .10 | .00 | .02 |
| Flexibility | -.03 | .15 | .08 | .17* | .15 | .05 |
| Coordination | -.14 | .03 | -.04 | .11 | -.11 | .04 |
| Health Behaviors (MHB-39) | .09 | .19* | -.12 | .16 | .08 | .10** |
| Safety | .09 | -.07 | -.07 | .10 | .05 | .04 |
| AWOL | .21* | .49*** | .23* | .30** | .34*** | .32*** |
| Compliance | -.08 | -.05 | -.17 | -.18* | -.18* | .09** |
| Diet | -.11 | .10 | .09 | .06 | .08 | .01 |
| Substance Consumption | -.03 | .06 | .14 | -.03 | -.04 | .03 |
| Hygiene | -.05 | -.12 | -.16 | -.03 | .01 | .03 |

*Note*. *N* = 128-141 for peer-ratings. SMAP = short measure of adult playfulness; OTD = Other-directed; LTH = Lighthearted; INT = Intellectual; WHI = Whimsical playfulness. CR-Fitness = Cardio-Respiratory Fitness. AWOL = Leading an active way of life.

*R*^2^ (OLIW) = Explained variance by all playfulness facets together, over the influence of sex and age.

**p* < .05. ***p* < .01. ****p* < .001. Two-tailed.
